# Exploiting ING2 Epigenetic Modulation as a Therapeutic Opportunity for Non-Small Cell Lung Cancer

**DOI:** 10.3390/cancers11101601

**Published:** 2019-10-21

**Authors:** Alice Blondel, Amine Benberghout, Rémy Pedeux, Charles Ricordel

**Affiliations:** 1 INSERM U1242, Chemistry Oncogenesis Stress and Signaling, CLCC Eugène Marquis, 35033 Rennes, France; alice.blondel@univ-rennes1.fr (A.B.); benberghout.amine@gmail.com (A.B.); remy.pedeux@univ-rennes1.fr (R.P.); 2 CHU Rennes, Service de Pneumologie, Université de Rennes 1, 35033 Rennes, France

**Keywords:** ING2, ING1, ING3, ING4, ING5, non-small cell lung cancer, NSCLC, mSin3A/HDAC complex, chromatin remodeling, HDAC inhibitors, therapeutic approach

## Abstract

Non-small cell lung cancer (NSCLC) has been the leading cause of cancer-related death worldwide, over the last few decades. Survival remains extremely poor in the metastatic setting and, consequently, innovative therapeutic strategies are urgently needed. Inhibitor of Growth Gene 2 (ING2) is a core component of the mSin3A/Histone deacetylases complex (HDAC), which controls the chromatin acetylation status and modulates gene transcription. This gene has been characterized as a tumor suppressor gene and its status in cancer has been scarcely explored. In this review, we focused on ING2 and other mSin3A/HDAC member statuses in NSCLC. Taking advantage of existing public databases and known pharmacological properties of HDAC inhibitors, finally, we proposed a therapeutic model based on an ING2 biomarker-guided strategy.

## 1. Introduction

Lung cancer is the leading cause of cancer-related death worldwide. Among the different subtypes of lung cancer, non-small cell lung cancer (NSCLC) is the prevailing subtype, accounting for about 80% to 85% of cases. The prognosis of NSCLC is poor with a 5-year survival rate of 15% [1]. Although great therapeutic advances have been made during the last decades, patients are often diagnosed with advanced stage disease, where only palliative chemotherapy or immunotherapy is recommended. In this context, deciphering biological relevance of tumor suppressor genes in cancer, such as the inhibitor of growth gene (*ING*) family, is an attractive approach to develop new therapeutic breakthrough. Among this protein family of chromatin readers, ING2 and ING1 allow the recruitment of mSin3A/HDAC chromatin remodeling complex on Histone H3 trimethylated on lysine 4 marks (H3K4me3) [2,3,4]. Histone deacetylases enzymes could promote adjacent histones deacetylation that would in turn result in chromatin remodeling and regulation of gene transcription, mainly inducing transcription repression [5]. In addition, as part of the p53 pathway, ING2 is involved in diverse cellular processes that are recognized as hallmarks of cancer [6,7,8] and its deletion in mice led to spontaneous soft tissue sarcomas formation [9]. ING2 is now characterized as a tumor suppressor gene and as such, its expression is frequently altered in human tumors. In this review, we assess literature and public databases to evaluate ING2 and other mSin3A/HDAC member statuses in NSCLC, in an effort to uncover new therapeutic opportunities.

## 2. ING2 Modulates Transcriptional Activity Through Chromatin Remodeling

The first member of the inhibitor of growth gene (*ING*) family, ING1, was initially discovered through an in vivo screen based on subtractive hybridization that aimed to identify tumor suppressor genes [10]. Subsequently, an in silico sequence homology search with ING1 allowed the identification of four other members of the ING family—ING2 [11,12], ING3 [13], ING4 and ING5 [14]. 

*ING* genes are made up of multiple exons, resulting in numerous transcribed variants, thanks to alternative mRNA splicing. The *ING2* gene is composed of three exons (1a, 1b, and 2) that can be alternatively spliced, thus, leading to two isoforms—ING2a and ING2b [15]. Using quantitative polymerase chain reaction (qPCR) to examine *ING2a* and *ING2b* expression level in different tissues, Unoki and colleagues found that both isoforms were ubiquitously expressed, albeit ING2a isoform expression was predominant. Moreover, as *ING2b* expression has only been detected at the RNA level and was never detected at the protein level, we focused this review on ING2a, which is thereafter referred to as ING2. 

The nucleosome, which is the fundamental chromatin subunit, consists of two pairs of each histones H2A, H2B, H3, and H4 with DNA wrapped around this octamer. The N-terminal tail of each histones, which emerges between the gyres of the DNA superhelix [16], contains highly conserved lysine residues that are the sites for various covalent modifications, including methylation [17]. These lysine methylations form binding sites for transcriptional regulator proteins [18]. More specifically, histone H3 trimethylated on lysine 4 (H3K4me3) has been reported to be exclusively associated with active transcription, while H3K4 dimethylated (H3K4me2) occurs at both inactive and active genes [19,20]. ING2 is able to bind to these marks of active transcription, with more affinity for H3K4me3 than for H3K4me2 [2].

**Figure 1:**
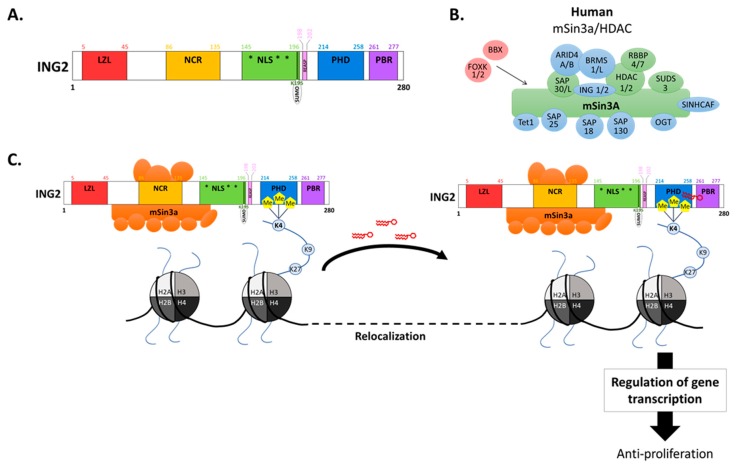
ING2 regulation of gene transcription through its interaction with H3K4me3 and the transcriptional regulator complex mSin3A/HDAC. (**A**) Protein structure of Human ING2. LZL—leucine zipper-like region; NCR—novel conserved region; NLS—nuclear localization signal, *within the NLS three short regions act as a nucleolar targeting signal (NTS); REASP—binding motif; PHD—plant homeodomain; PBR—polybasic region. ING2 structure was built according to UniProtKB ING2_Human (Q9H160). (**B**) Mammalian Sin3A/HDAC complex members. The core Sin3A subunits are depicted in green, the Sin3A associated proteins are depicted in blue, and the transcription factors are depicted in red. The names given for each complex member is the one approved by the HUGO Gene Nomenclature Committee (HGNC). (**C**) Schematic representation of ING2/H3K4me3/Sin3A formation regulating gene transcription. ING2 PHD domain recognizes trimethylated H3K4 (H3K4me3) as well as phosphatidylinositol 5-phosphate (PI(5)P) while the ING2 N-terminal part is detected by the transcriptional regulator complex mSin3-histone deacetylase. The ING2 sumoylation at Lysine 195 increases its association with this complex. An elevation in PI(5)P nuclear level triggers ING2/mSin3A complex relocalization to novel chromatin sites to regulate the transcription of target genes.

The biological roles of ING2 are related to its various domains (Figure 1, panel A) and more particularly, to its plant homeodomain (PHD), which is characterized by a Cys^4^-His-Cys^3^ zinc-binding motif that allows ING2 stabilization at active chromatin, through the binding to H3K4me3 [2,3]. The PHD motif of ING2 acts as a dual-specificity module that binds to phosphatidylinositol 5-phosphate (PI(5)P) [21], in addition to H3K4me3. PI(5)P also requires the polybasic region (PBR) that is located immediately after the PHD domain (Figure 1, panel A) to bind efficiently to ING2 [22] and this binding is suggested to change the ING2 sub-nuclear distribution, in order to localize it at target gene promoters [23]. This targeting is crucial for recruiting ING2-associated HDAC activity to target gene promoters. Indeed, ING2 is part of the mSin3A-HDAC complex [4], thanks to its interaction with SAP30, mSin3A, and HDAC1 [24]. This interaction is due to its 40–140 N-terminal motif [25], which is involved in chromatin remodeling. Depicting all the mSin3A/HDAC complex members illustrates this mechanism (Figure 1, panel B). Indeed, this multiprotein complex with mSin3A being its core component, is associated with HDAC 1 and 2 [26], that constitutes the major catalytic subunits. An additional core mSin3A/HDAC protein, AT-rich interactive domain-containing protein 4B (ARID4B), is believed to function as a linker between the mSin3A/HDAC complex and the nucleosome, thus, stabilizing their interaction [27]. Some other members of the complex are involved in the recruitment of the HDAC activity, such as BRMS1/L or SAP30/L [28,29], whereas factors as SIN3A Corepressor Complex Component (SUDS3) [30] and O-linked N-acetylglucosamine transferase (OGT) [31] specifically stabilizes HDAC within the complex, while Sin3A Associated Protein 18 (SAP18) [26] and SIN3-HDAC Complex Associated Factor (SINHCAF) [32] help tethering the complex to the target gene promoter, thereby allowing HDAC to regulate gene transcription (Figure 1, panel C). Finally, SAP130 enables the modulation of mSin3A/HDAC transcriptional repression activity by binding a coactivator [33]. Of note, it has been shown that the sumoylation of ING2 at Lysine 195 enhances ING2 association with the mSin3A/HDAC complex [25]. As this lysine residue belongs to a phosphorylation-dependent SUMO modification (PDSM) consensus sequence, some authors suggest phosphorylation could modulate this interaction [25], but it remains to be demonstrated experimentally.

Altogether, mSin3A/HDAC chromatin remodeling complex has been originally reported to massively repress the transcription of a number of genes [5,24,34,35]. Nevertheless, the same complex was recently shown to activate some genes [5,24,36], although this capacity seems limited to a small number of genes. Indeed, in a study based on the comparison of gene expression levels in wild-type versus SIN3-deficient cells in *Drosophila* using full-genome oligonucleotide microarrays, a 10-fold difference was found between the number of activated and repressed genes [5]. Hence, ING2 functions as a bridge to link mSin3A/HDAC complex to H3K4me3, thereby, promoting the deacetylation of adjacent acetylated histone residues, which in turn allows chromatin remodeling and regulation of gene transcription (Figure 1, panel C). Of note, ING1 and ING2 are mutually exclusive in the mSin3A/HDAC complex [37] and the binding affinity for H3K4me3 is greater for ING2 compared to the one of ING1 [3].

Another key role for ING2 involves the p53 pathway as ING2 promoter contains two p53 binding sites [38]. Furthermore, the expression of ING2 results in p53 acetylation at Lysine 382 through the histone acetyltransferase p300 [12]. This enhanced acetylation led to p53 activation, ultimately preventing cell proliferation through induction of either senescence [39], apoptosis [40,41], or cell-cycle arrest in G1 [42]. Hence, ING2 is involved in multiple cellular processes, most of which are recognized hallmarks of tumorigenesis (cell-cycle regulation, replicative senescence [7], DNA repair [8], and DNA replication [6]). Moreover, a study conducted on *ING2* knockout mice indicates that ING2 deficiency spontaneously increases soft tissue sarcoma formation [9]. 

All of these facts highlight *ING2* as a tumor suppressor gene, playing a critical role against tumor development and cancer, notably through the regulation of mSin3A/HDAC-mediated epigenetic functions.

## 3. ING2 Status in Human Tumors

### 3.1. ING2 Alterations in Human Tumors

As a tumor suppressor gene, several studies have explored *ING2* gene status in different types of human tumors, whether at the genomic, transcriptomic or protein level. Using immunohistochemistry assay, it has been reported that ING2 protein expression was decreased in patients with, melanoma, breast cancer, hepatocellular carcinoma (16/29 and 44/84) and osteosarcoma [43,44,45]. This downregulation was correlated with tumor size, histological and pathological classification, alpha-fetoprotein serum level, and the overall survival in hepatocellular carcinoma [44], whereas it only correlated with the overall survival in patients with osteosarcoma [46]. Conversely, overexpression of the ING2 protein was found in patients with endometrial carcinoma and hepatocellular carcinoma (40/84) [44,45]. Moreover, studies on tumor cell lines also found that ING2 was overexpressed in cervical carcinoma, colon cancer, and acute lymphoblastic leukemia (ALL) cell lines [36,45,47]. However, the precise mechanism by which *ING2* expression is altered was not fully elucidated in most studies. Nevertheless, some authors found that miR-153-3p represses *ING2* expression in ALL cells by binding to the 3′-UTR site, and that miR-153-3p is downregulated in ALL cells [47], leading to *ING2* overexpression. Additionally, NF-kB can bind to the *ING2* promoter region and activate *ING2* transcription in colon cancer. Finally, other authors have suggested that activation of NF-kB lead to the upregulation of ING2 [36].

At the transcriptomic level, studies found that ING2 mRNA was downregulated in several tumor types including basal cell carcinoma (75/75), hepatocellular carcinoma and osteosarcoma [44,46,48]. *ING2* downregulation has also been reported in breast, lung, ovarian, pancreatic, and prostate cancer cell lines [49,50]. On one hand, *ING2* upregulation at the mRNA level was reported in patients with colon cancer (18/34) [36], even if the underlying mechanism was not investigated in this study. On the other hand, one publication suggested that the *ING2* gene could be targeted on its 3′-UTR site by miR-8084, which showed tumor-promoting properties in breast cancer [51]. This post-translational regulation of the *ING2* gene expression might explain the discrepancies observed in many publications between *ING2* mRNA level and ING2 protein expression [52]. 

Finally, at the genomic level, loss of heterozygosity (LOH) of the ING2 chromosomal region has been reported in ameloblastoma (14/28), head and neck squamous cell carcinoma (HNSCC) (30/55) (4q35.1), hepatocellular carcinoma (41.2%) (4q34–35.2), and basal cell carcinoma (3/11) (4q32-35) [53,54,55,56,57]. Moreover, LOH of the 4q35.1 region in HNSCC was correlated with tumor stage and disease-free survival but not with node status or overall survival [54], whereas no correlation has been found in hepatocellular carcinoma [55]. Additionally, deletion of the chromosomic region 4q34–35.2 containing the *ING2* gene has also been reported in uterine leiomyosarcoma (3/6) [58], and hypermethylation of the seven CpG sites in multiple intronic regions of the *ING2* gene has been shown in patients with esophageal squamous cell carcinoma. This was also observed in matched plasma cell-free DNA samples [59], even if the biological consequence of this intronic methylation on *ING2* expression remains unknown.

**Figure 2:**
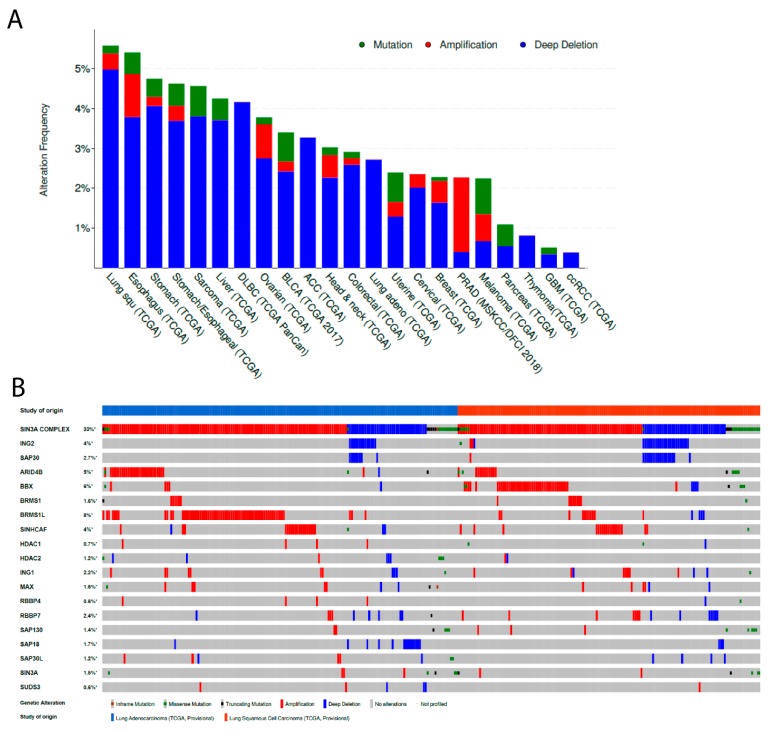
Despite *ING2* being rarely altered at the genomic level in cancers, genomic alteration of at least one member of the mSin3A/HDAC complex is frequent. (**A**) Bar graph showing the alteration frequency according to pathology (from the TCGA database). Blue represents gene deletion, red represents gene amplification, and green represents gene mutation. (**B**) Heatmap representing genomic alterations of mSin3A/HDAC members, according to NSCLC subtype (adenocarcinoma or squamous cell carcinoma) (from the TCGA database). First line is a pool of all mSin3A/HDAC member genomic alterations. Of note, specimens without any genomic alteration concerning the mSin3A/HDAC members are not depicted in the figure.

To explore further *ING2* status in cancer, we used The Cancer Genome Atlas database [60,61] to analyze alterations of the *ING2* gene and the members of the mSin3A/HDAC complex. This analysis revealed that 221 out of 9,892 (2.23%) human cancer samples included in the TCGA Pancancer study presented an alteration of *ING2* gene and 161 of these 221 (73%) alterations were deletions. Moreover, 2,384 out of 9,892 (24.1%) tumor samples presented an alteration in in at least one member of the Sin3a HDAC complex. Interestingly, one of the tumor types with the most frequent alteration in ING2 was the lung squamous cell carcinoma, comprising 5.54% of the tumors involved (Figure 2, panel A).

### 3.2. ING2 Alterations in Non-Small Cell Lung Cancer

ING2 has also been reported to be altered in NSCLC at the protein, transcriptomic, and genomic level. Indeed, two independent studies showed that ING2 expression levels were decreased at the protein level (independent of the p53 status). A study observed the loss of *ING2* expression in 70 out of 120 (58.3%) NSCLC [62] (Table 1), and was more frequent in adenocarcinoma than in squamous cell carcinoma (68% and 45%, respectively). However, no correlation was observed between ING2 expression in NSCLC and age, gender, disease stage, or patient survival [62]. Another study, conducted on Chinese patient specimens, showed that the ING2 expression was lost in 21 of 64 (32.8%) NSCLC and more frequently in ADK than in SCC (45.8% and 26.3%, respectively) (Table 1). ING2 loss was correlated with lymph node metastasis status and TNM stage in squamous cell carcinoma, but not in adenocarcinome [63]. Whether these discrepancies between these two studies are due to antibody specificity or ethnic disparities (notably, oncogenic driver mutational status was unknown in both studies), remains to be elucidated.

Moreover, some study supports a transcriptional control of *ING2* expression in NSCLC showing a correlation with *ING2* mRNA decrease and low protein expression [62]. Consistently, studies also reported that ING2 was downregulated at the mRNA level in lung cancer cell lines [49,50]. Nevertheless, contradictory results have been found when analyzing TCGA and The Human Protein Atlas (THPA) databases, as the authors did not find any correlation between INGs mRNA and protein expression [52]. It is worth noticing that ING2 degradation has been shown to be mediated by Smad 1 ubiquitination regulatory factor 1 (Smurf 1) [65], a protein highly expressed in lung cancer [66]. Therefore, this mechanism could participate to the contradictory observation made between ING2 mRNA level and protein expression in lung cancer.

Another alteration frequently occurring in NSCLC is the chromosomal deletion of the 4q35.1 region, which includes *ING2* gene. Indeed, it has been reported that the chromosomal region 4q34.2–35.1 was deleted in 2 out of 10 patients with NSCLC [64] (Table 1). However, further investigations on larger samples of patients need to be performed to confirm this observation. Additionally, this chromosomal region contains not only *ING2* gene but also many other genes such as *SAP30* and micro-RNAs (miR-6082; miR-548T; miR-4276; miR-1305; miR-3945; and miR-4455). Therefore, it is still unclear if the deletion of *ING2* alone or the whole chromosomic region is implicated in promoting tumorigenesis. Concerning *ING2* mutations, only silent ones have been found in NSCLC. As an example, a study reported a substitution (C to T) in exon 1 at codon 13 in 6 out of 31 lung cancer (without any change of the encoded amino acid (Alanine) [49]) was likely to be a polymorphism (Table 1). 

Interestingly, the analysis of the mSin3A/HDAC complex member alterations in NSCLC via the TCGA database revealed that *ING2* was altered in 20 out of 408 (4.9%) NSCLC and was found to be higher in squamous cell carcinoma than in adenocarcinoma (6.7% and 2.6%, respectively) (Figure 2, panel A). Moreover, the *ING2* gene deep deletion was the most frequent alteration with 17 out of 408 (3.54%) NSCLC and was found to be higher in squamous cell carcinoma than in adenocarcinoma (6.18% and 2.61%, respectively). Only one missense mutation (E204Q) of *ING2* of unknown significance was reported out of the 408 NSCLC samples. Focusing on all members of the mSin3A/HDAC complex, data revealed that 152 of the 408 (37.3%) NSCLC presented an alteration in at least one member of the mSin3A/HDAC complex, occurring in 38.8% of squamous cell carcinoma specimens and 36.1% of adenocarcinoma specimens (Figure 2, panel B). Notably, we found that patients presenting an alteration in at least one member of the mSin3A/HDAC complex had a higher probability of prolonged overall survival, in comparison with patients without any alteration of the complex in lung squamous cell carcinoma (*p* = 0.0451) (data not shown).

In summary, the tumor suppressor protein ING2 was found to be altered in human tumors, especially in NSCLC, mainly at the protein level (varying from 32.8% to 58.3% according to different studies). *ING2* gene alterations in NSCLC are predominantly deletion, but still remain a rare event (less than 3%). Strikingly, genomic alteration of at least one component of the mSin3A/HDAC complex appears to be relatively common in human cancers and NSCLC (24.1% and 33%, respectively) (Figure 2, panel B). 

## 4. Potential Role of the ING2 Epigenetic Modulation in Lung Cancer Treatment

NSCLC is a deadly disease and the leading cause of cancer-related death worldwide. Even if great advances in the field have been made during the last decade, notably thanks to the emergence of targeted therapies and immunotherapies, survival remains poor in the metastatic setting. In this context, developing new therapeutic approaches based on predictive biomarkers is still an active area of research [67]. As discussed above, ING2 genomic alterations are rare in non-small cell lung cancer, and loss at the protein level has been reported within a large proportion range. Even though the number of studies that investigate the expression of ING2 protein are limited in lung cancer, its potential use as a diagnosis biomarker has been suggested by some authors [68].

### 4.1. Exploring ING2 Dependencies in Cancer Cells

**Figure 3:**
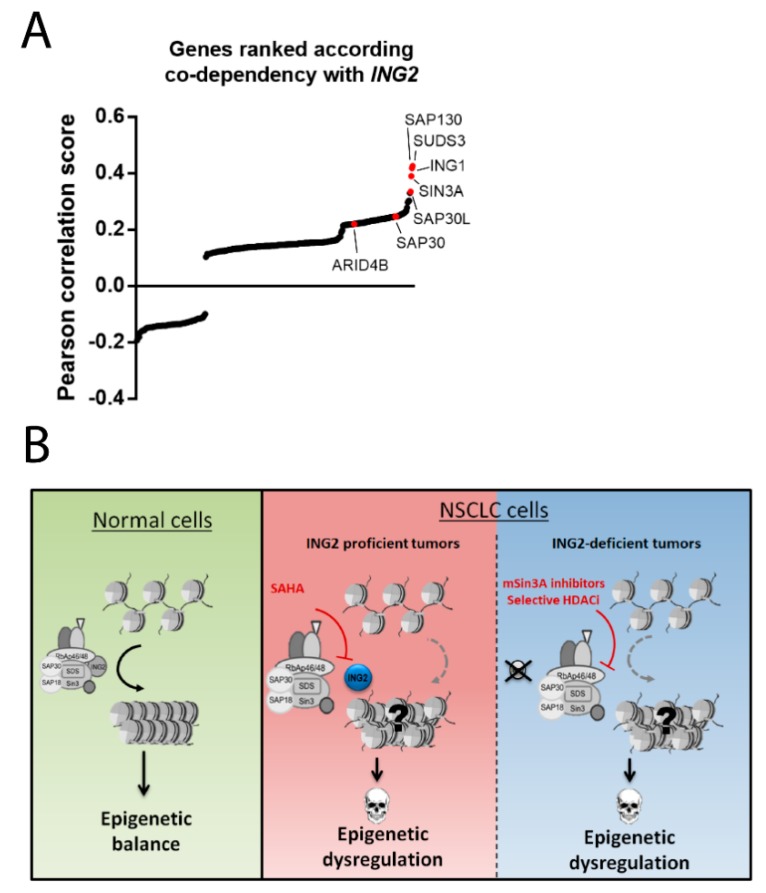
Co-dependency between *ING2* and mSin3A/HDAC complex members in tumor cell lines. (**A**) Graph depicting ranked Pearson correlation score between the CERES dependency score for each tested gene in the Cancer Dependency Map Project and the ING2 CERES dependency score. (**B**) Working model for a ING2 biomarker-based therapeutic strategy in NSCLC. Tumors expressing ING2 are more likely to depend on the oncogenic properties of mSin3A/HDAC for survival and could be targeted by suberoyl anilide hydroxamic acid (SAHA). Tumors that lose ING2 expression cannot be treated by SAHA, but can be treated by mSin3A direct inhibitors (mSin3Ai) or HDAC1/2 inhibitors (s.HDACi).

Recently, lot of great efforts have been made to uncover genetic vulnerabilities in cancer, notably taking advantage of the emergent CrispR-screening methods. The Cancer Dependency Map project, led by the Broad Institute, is a systematic high-throughput screening of genotype-specific cancer vulnerabilities in tumor cell lines [69]. According to the latest dataset (CrispR AVANA public 19Q2), many mSin3A/HDAC members are found among the top *ING2* gene co-dependencies in cancer cell lines (Figure 3, panel A). These results suggest that many tumor cells lines are dependent on a functionally active mSin3A/HDAC complex for proliferation. Consequently, we speculate that it could be utilized as an “Achilles’ heel”, potentially leading to a biomarker-guided therapeutic model (Figure 3, panel B).

Moreover, multiple studies support the important biological role of the mSin3A-HDAC complex in tumors. Of note, it was originally shown that mSin3A/B recruitment was necessary to antagonize c-MYC induced transcriptional activation [70]. *SIN3A*-knockout studies using multiples physiological models demonstrate that a reduction of the mSin3A levels correlates with a loss of proliferative abilities [71,72]. Interestingly, *mSin3A* loss of expression in mouse foregut endoderm lead to severe defect in lung morphogenesis and development [73], underlying its biological role in lung epithelial cells. In cancer cells, whether the mSin3A/HDAC complex as a whole show more tumor suppressive or oncogenic properties, is still under debate [74]. However, the relative high frequencies of genomic alteration concerning at least one member of the mSin3A/HDAC complex in NSCLC TCGA samples, notably gene amplification (23.6% of adenocarcinoma and 17.53% of squamous cell carcinoma) (Figure 2, Panel B), clearly pledges for a “gain-of-function” phenotype predisposing tumors cells to a selective advantage. Additionally, small molecule inhibitors targeting Paired Amphipathic Helix 2 (PAH2) domain of mSin3A was shown to induce transcriptional reprogramming, impairment of clonogenic abilities, inhibition of proliferation, and metastasis in triple negative breast cancer models [75]. Coherently, the Cancer Dependency Map project database characterizes *SIN3A* gene as a “common essential” gene in a vast majority of cancer cell lines. It is of note that *SIN3A*-knockout mice embryos stop developing after 6.5 weeks [76]. Consequently, targeting mSin3A itself, or specific partners of the complex, appears to be an attractive therapeutic strategy in cancer. Conversely, it is hard to conciliate the fact that ING2 is a recognized tumor suppressor protein belonging to a multi-protein complex that tends to proffer oncogenic properties in cancer cells. However, some could argue that many non-epigenetic functions of ING2 favor its tumor suppressive activity (DNA repair, DNA replication, cell cycle progression, etc.) but it still remains an unresolved issue to date.

### 4.2. Targeting SIN3A-Mediated ING2 Functions in Cancer Cells

Suberoyl anilide hydroxamic acid (SAHA), also called vorinostat, a class I, II, and IV HDAC inhibitor (HDACi), was described as a potent inhibitor of the mSin3A/HDAC complex-mediated functions [37]. More precisely, ING2 protein interaction with the mSin3A-HDAC complex was abrogated in HEK 293T cells treated with SAHA [77]. Interestingly, HDAC inhibitor induced dissociation of ING2 with the mSin3A/HDAC complex was independent from the PHD domain of ING2, suggesting that this drug effect was not dependent on the ING2 chromatin fixation. Similar observations were made with other HDACi molecules (trichostatin A and apicidin). Notably, ING2 and mSin3A/HDAC complex occupancy on the tumor suppressor *p21* promoter was reduced in SAHA treated HEK 293T. Consequently, authors hypothesized that ING2 disruption lead to the induction of *p21* transcription, as it was observed, upon HDACi treatment [78]. Conversely, an independent study showed that *p21* expression was reduced in ING2-downregulated U2OS cells, leading to cell cycle progression, in a p53-independent manner [79]. These contradictory findings might be explained by the broader cellular effects of SAHA, a pan-HDAC inhibitor [80], or by the potential impact of SAHA on ING2 post-translational modification. Notably, as already mentioned above, ING2 sumoylation on Lysine 195 has been described to enhance its interaction with mSin3A [25]. Therefore, whether ING2 disruption induced by SAHA is dependent on its sumoylation status is an interesting question that needs to be explored. Nevertheless, the use of SAHA represents an attractive approach to target ING2-mediated mSin3A/HDAC epigenetic functions.

Interestingly, the Cancer Dependency Map also uncovered a co-dependency between ING1 and ING2 in multiple cancer cell lines (data not shown) suggesting a potential crosstalk between the protein’s functions. Coherently, these two members of the ING family share an important structural homology, except for the N-terminal part, and are both stable and exclusive components of the mSin3A/HDAC complex. However, the subscription of ING1 and ING2 to this complex is highly conserved throughout evolution [81], suggesting that some ING1 and ING2 mSin3A/HDAC-mediated functions are biologically important but not redundant. In line with this hypothesis, *ING1* and *ING2* double knock-out mice show embryonic lethality (not published), but not their single knock-out counterparts, which shows distinct phenotypes [9,82]. In terms of therapeutic approach, HDAC inhibitors do not disturb ING1 interaction to mSin3A/HDAC [77], and it is, therefore, unknown if HDAC inhibitors could impact ING1 epigenetic functions. Finally, to our knowledge no study to date has concomitantly explored the status of ING1 and ING2 proteins in human cancers. Altogether, more experimental data are needed to precisely decipher the potential redundant or exclusive mSin3A/HDAC-mediated roles relying on ING1 and ING2. 

During the last two decades, many HDAC inhibitors have been developed and were tested in clinical trials due to their pleiotropic antineoplastic properties [83]. Indeed, in various types of cancer, histone deacetylation was found to deeply impact cellular fate [84]. Of note, studies claiming to inactivate mSin3A as a therapeutic target in leukemia are limited to the use of non-selective HDAC inhibitors that do not target mSin3A directly. To date, HDAC inhibitors have only been approved for T cell-lymphoma (vorinostat, romidepsin, and belinostat) and multiple myeloma (panobinostat), but many clinical trials are ongoing in solid tumors [85]. As the specificity of HDAC inhibitors have greatly improved over the last decades, deciphering the biological consequences of these treatments on ING2 epigenetic function is of prime interest in order to explore new therapeutic strategies.

Altogether, these findings lead us to hypothesize a biomarker-based therapeutic strategy, involving ING2 as a tumor predictive factor for SAHA sensitivity in NSCLC (Figure 3, Panel B). In light of the observation of a high frequency of mSin3A/HDAC complex-alteration in cancer, determining if such genomic alteration could make ING2-expressing cells more prone to HDAC inhibitors efficacy is a very interesting hypothesis to elucidate. Conversely, in ING2-non-expressing cells, as this “target” could not be disrupted by SAHA, small molecules that directly inhibit mSin3A or more specific HDAC inhibitors, could represent a more convenient way to circumvent this issue. Experimental studies are actually ongoing to explore this working hypothesis.

## 5. Conclusions

ING2 protein is a chromatin reader and a stable component of the mSin3A/HDAC complex. Several tumor suppressive functions have been described for ING2 in cancer cell lines, and in accordance with these observations, ING2 expression is lost in many cancer subtypes. Interestingly, genomic alterations of mSin3A/HDAC members occur frequently in NSCLC, mainly through gene amplification. Recent experimental data have revealed that a vast majority of tumor cell lines depend on a functionally active mSin3A/HDAC complex to proliferate. As ING2 can be pharmacologically disrupted from the mSin3A/HDAC complex by SAHA, we built a model based on an ING2 biomarker-guided therapeutic hypothesis. Further research is still warranted to confirm the predictive value of ING2 in clinical practice of NSCLC.

## Figures and Tables

**Table 1:** ING2a status in human lung cancer.

Tissue Type	Origin	Mutation Type/Expression Change	Methods	Position	Coding	Frequency	Ref.
Lung cancer	Cell lines	Downregulation	RT-QPCR			7/8	[49]
	Patient	Substitution	PCR-SSCP, Sequencing	LZL (13)	Ala -> Ala	6/31	
	Patient	Substitution	PCR-SSCP, Sequencing	6bp downstream exon 1		6/31	
Lung cancer	Cell lines	Downregulation	Q-PCR			2/2	[50]
Lung cancer	Patient	Downregulation	IHC			70/120	[62]
	Patient	No LOH	MM			0/12	
	Patient	Substitution	Sequencing	39	Ala -> Ala	21/22	
	Patient	Downregulation	Q-PCR			15/22	
		No change	Q-PCR			6/22	
		Upregulation	Q-PCR			1/22	
NSCLC	Patient	Downregulation, aberrantly localization	IHC, RT-PCR, WB			21/64 (32.8%)	[63]
Adenocarcinoma	Patient	Downregulation, aberrantly localization	IHC, RT-PCR, WB			11/24 (45.8%)	
Squamous cell carcinoma	Patient	Downregulation, aberrantly localization	IHC, RT-PCR, WB			10/38 (26.3%)	
NSCLC	Patient	Chromosomal deletion	cDNA Microarray	4q34.2–q35.1		2/10 (20%)	[64]

Abbreviations: IHC—Immunohistochemistry; LOH—Loss of Heterozygosity; LZL—Leucine Zipper Like domain; MM—Microsatellite Marker; NSCLC—Non Small Cell Lung Carcinoma; PCR–SSCP—Polymerase Chain Reaction–Single Strand Conformation Polymorphism; RT–PCR—Retro transcription–Polymerase Chain Reaction; Q-PCR—Quantitative-Polymerase Chain Reaction; and WB—Western Blot.
